# Was the killing and forced sterilization of psychiatric patients in nazi Germany a genocide? What is the relevance of this tragedy today?

**DOI:** 10.1192/j.eurpsy.2026.10977

**Published:** 2026-07-31

**Authors:** E. Thys

**Affiliations:** Psychiatry, UPC KU Leuven, Leuven, Belgium

## Abstract

**Introduction:**

The 1948 Genocide Convention (GC) defines genocide as the intentional killing of groups of people, including forced sterilization. This presentation explores whether the Nazi regime’s forced sterilization and mass killing of neuropsychiatric patients, involving over 400,000 sterilizations and 300,000 deaths, qualifies as genocide under the GC. The relevance of this approach for psychiatry today is also discussed.

**Objectives:**

To explore, from a legal point of view, whether the Nazi atrocities qualify as genocide under the GC. To discuss the relevance of this approach for psychiatry and society today.

**Methods:**

To examine and compare: the historical facts from nazi Germany, the GC and the scientific literature, and the international jurisprudence concerning genocide, more specifically the International Criminal Tribunal for the former Yugoslavia (ICTY), the International Criminal Tribunal for Rwanda (ICTR) and the International Criminal Court’s (ICC) dealing with the situation in Darfur.

**Results:**

The GC, originated after the Second World War and the Holocaust, and ratified by 153 nations, has great importance. But its definition of the “protected groups” (the victims) proves to be problematic in the three modern day genocide trials (ICTY, ICTR and ICC-Darfur). This has to do with its four, exhaustive categories: religious, ethnic, national and racial groups. Although from a current point of view, the patients don’t belong to these groups, they were clearly considered as a racial group by the perpetrators. Moreover, in recent publications, the German Bundestag recognizes the victims as a “racially persecuted group.”

**Conclusions:**

We conclude that the mass murder and forced sterilization of psychiatric patients in nazi Germany can be considered a genocide. The GC being too recent, it can not prosecute the perpetrators. But the theoretical recognition holds great relevance for today’s patients:

1. Commemoration and Rehabilitation: The victims of these atrocities have received inadequate memorialization.
2. Education and Prevention: Recognizing this genocide would serve as a warning and educational tool against the ongoing stigmatization and discrimination.
3. Historical Significance and Naming: Officially acknowledging these events as genocide would validate their historical significance and strengthen efforts to prevent future abuses. The proposed name “Psychogenocide,” would reflect the nature of the crimes.

Recognizing this genocide would promote further research, help combat the stigma faced by individuals with psychiatric and physical disabilities, and serve as a global precedent for preventing similar atrocities.

**Disclosure of Interest:**

None Declared

